# Completed audit cycle to explore the use of the STOPP/START toolkit to optimise medication in psychiatric in-patients with dementia

**DOI:** 10.1192/bjb.2017.10

**Published:** 2018-02

**Authors:** Victor M. Aziz, Natalie Hill, Sugandha Kumar

**Affiliations:** 1 Cynon Valley Hospital, Wales; 2 Wales Deanery, Cardiff

## Abstract

**Aims and method:**

To explore the use of the STOPP/START toolkit in older psychiatric in-patients with dementia. Clinical records and current drug charts were reviewed against STOPP/START criteria for all in-patients (*n* = 86) on six specialist dementia wards.

**Results:**

Benzodiazepines, antipsychotics and opiates were most commonly prescribed inappropriately. The most common unprescribed medication groups were statins, calcium supplements and vitamin D supplements. There was an overall reduction of 7% in comorbidities and 8% in the number of prescriptions. *t*-test showed a significant drop in average comorbidities between both audits, *t*(1) = 23.920, *P* = 0.027, and in average prescriptions per patient, *t*(1) = 28.808, *P* = 0.022. There was no difference in the number of patients receiving polypharmacy, *t*(1) = 7.500, *P* = 0.084, or receiving medication with a high risk of adverse drug reactions, *t*(1) = 6.857, *P* = 0.092.

**Clinical implications:**

The STOPP/START toolkit highlighted the importance of collaborative working between doctors, clinical pharmacists and nursing staff, and could provide old age psychiatrists with a structured tool to identify inappropriate prescribing of non-psychiatric medications.

**Declaration of interests:**

None.

There are 10 million people in the UK over the age of 65. The latest projections suggest there will be an additional 5.5 million older people by 2036, and that the current number will have nearly doubled to around 19 million by 2050. Older people have a high prevalence of chronic and multiple illnesses and are likely to be prescribed multiple medications. Potentially inappropriate prescribing (PIP) is reported to be highly prevalent in this age group, and has been associated with adverse drug events (ADEs) leading to admission to hospital and death.^1^ Inappropriate prescribing occurs when the risks associated with prescribing a medication outweigh the potential benefits of the medication in a particular patient. PIP may also occur when a patient does not receive a medication indicated for the treatment or prevention of a disease or condition.^2^ Pharmacokinetics and pharmacodynamics may be altered by ageing or disease. This puts older people at a high risk of adverse drug reactions (ADRs), ADEs and drug–drug interactions.

Some of the drugs that are considered a high risk with respect to hospital admissions include: non-steroidal anti-inflammatory drugs (NSAIDs, including aspirin), diuretics, warfarin, angiotensin-converting enzyme inhibitors (ACEIs)/angiotensin-II receptor antagonists (A2RAs), antidepressants, lithium, beta-blockers, opiates, digoxin, prednisolone and clopidogrel.^3^

The National Service Framework (NSF) for older people recommends that an older patient should have medication reviews to reduce medicine-related problems. Studies in general practices and care homes have demonstrated that pharmacists undertaking medication reviews can improve the quality of care, optimise the use of medicines and produce cost-effective savings.^4^^,^^5^

The aim of the audit was to review all prescribed medication in psychiatric in-patients over the age of 65 with a diagnosis of dementia in the Cwm Taf University Health Board (UHB) area covering a population of 300,000, to determine the degree of inappropriate prescribing and to optimise medication.

## Methodology

Cwm Taf UHB is responsible for providing healthcare services (hospital- and community-based services) to the population of Merthyr Tydfil and Rhondda Cynon Taf, estimated to be around 289 400 people. The Health Board is divided geographically into four sectors.

The audit included all mental health patients with a diagnosis of dementia who were in-patients on 1 December 2015. All psychiatric assessment wards (for those over the age of 65) and specialist dementia wards in Cwm Taf UHB were audited. We identified 47 patients in December 2015 and 39 patients in the re-audit in May 2016. A thorough past medical history and current medication history were established for each patient using the clinical records. All diagnoses were made using internationally agreed standard criteria, such as the National Institute of Neurological and Communicative Disorders and Stroke and the Alzheimer's Disease and Related Disorders Association criteria, by the consultant psychiatrist and their teams in either the community or in-patient settings. The dementia ranged from moderate to severe for all specialist dementia wards. For the purpose of this audit, polypharmacy was defined as more than five medications. The audit used the second version of the toolkit;^6^ the tool itself takes about 30 min to complete.

Some tools have been developed to identify older people at risk from adverse effects and to reduce the risk of initiating drugs likely to cause adverse events. These include the screening tool of older persons’ potentially inappropriate prescriptions (STOPP) and the screening tool to alert doctors to the right treatment (START). The toolkit was not designed for mental health patients. However, the STOPP–START tool has been validated in patients aged 65 and over by a consensus panel of 18 experts in geriatric pharmacotherapy in Ireland and the UK.^7^^,^^8^^,^^9^ The panel included experts in geriatric medicine, clinical pharmacology, old age psychiatry, clinical pharmacy and primary care medicine. Interrater reliability of the STOPP (K = 0.75) and START (K = 0.68) criteria was tested in six different European countries.^8^ A further study^10^ found higher interrater reliability of STOPP (K = 0.97) and START (K = 0.92).

All prescribed medication was checked against the STOPP/START criteria. In the STOPP/START tool, STOPP comprises 65 clinically significant criteria for PIP in older people. Each criterion is accompanied by a concise explanation as to why the prescribing practice is potentially inappropriate. It emphasises potential adverse drug–drug interactions and duplicate drug class prescriptions. An example is the advice to stop NSAIDs in patients with a history of peptic ulcer disease or gastrointestinal bleeding because of the risk of peptic ulcer relapse. In addition, NSAIDs should be stopped with moderate to severe hypertension (moderate: 160/100–179/109 mmHg; severe: ≥180/110 mmHg) because of the risk of exacerbation of hypertension.

START consists of 22 evidence-based criteria and identifies potential prescribing omissions (PPOs), e.g. start levodopa in idiopathic Parkinson's disease with definite functional impairment and resultant disability.

A *t*-test was conducted to compare the means between the initial audit and the re-audit. Following the initial data analysis, the audit was presented to all involved teams and action plans were introduced.

### Agreed action plan following the first audit

It was agreed that all teams would regularly review the medication charts along with the clinical pharmacist to optimise prescribing. Small pocket-sized smartcards/leaflets showing the drugs commonly meeting the STOPP/START criteria were also produced. Smartcards were placed alongside all medication charts on the wards. Training regarding the use of the STOPP/START toolkit was also incorporated into team inductions for junior doctors and nursing staff. A re-audit was completed on 1 May 2016 to determine the impact of these changes.

## Results

Table 1 shows a summary of the demographics across both audit cycles. There was no significant difference in age between the two audits. According to the Mann–Whitney U test, the distribution of the female gender was the same across both audits, *P* = 1.000. There was no significant difference in the proportion of patients on specialist dementia wards in the two audits. There was no reduction of medications prescribed in the community for patients on the acute wards before those patients came into hospital.

**Table 1:** Demographic for all sectors

	First audit	Re-audit	
Number of patients	58	47	
Age, years (mean ± s.d.)	78.33 ± 2.74	78.72 ± 3.11	
Female gender (%)	31 (53.5)	29 (63)	
Diagnosis (*n*): AD VAD Dementia with Lewy bodies Parkinson's disease/dementia Head injury Mixed Unspecified	18 21 5 – 1 7 6	20 15 6 1 – 3 2	
Average comorbidities per patient (mean ± s.d.)	6.23 ± 1.52	5.73 ± 1.02	*t*(1) = 23.920, *P* = 0.027
			95% CI = 2.803–9.157
Average number of prescriptions per patient (mean ± s.d.)	10.88 ± 1.27	10.15 ± 0.58	*t*(1) = 28.808, *P* = 0.022
			95% CI = 5.877–15.153
Number of patients receiving polypharmacy (%)	51 (88)	39 (85)	*t*(1) = 7.500, *P* = 0.084
			95% CI = −31.237–121.237
Number of patients receiving medication with high risk of adverse drug reactions (%)	55 (95)	41 (89)	*t*(1) = 6.857, *P* = 0.092 95% CI = −40.943–136.943
Number of patients on specialist dementia wards (%)	48 (83)	39 (83)	*t*(1) = 9.667, *P* = 0.066
			95% CI = −13.678–100.678

There was an overall 7% reduction in the number of comorbidities and an 8% reduction in the number of prescriptions. The *t*-test showed a significant drop in the average number of comorbidities between the two audits, *t*(1) = 23.920, *P* = 0.027, and a drop in the average number of prescriptions per patient, *t*(1) = 28.808, *P* = 0.022. The overall improvement in prescribing contributed to a 19% reduction in bed occupancy for specialist dementia beds.

However, there was no difference in the number of patients receiving polypharmacy, *t*(1) = 7.500, *P* = 0.084, or the number of patients receiving medication with a high risk of ADRs, *t*(1) = 6.857, *P* = 0.092. There was also no significant difference in the number of patients in specialist dementia wards, *t*(1) = 9.667, *P* = 0.066.

**Fig. 1:**
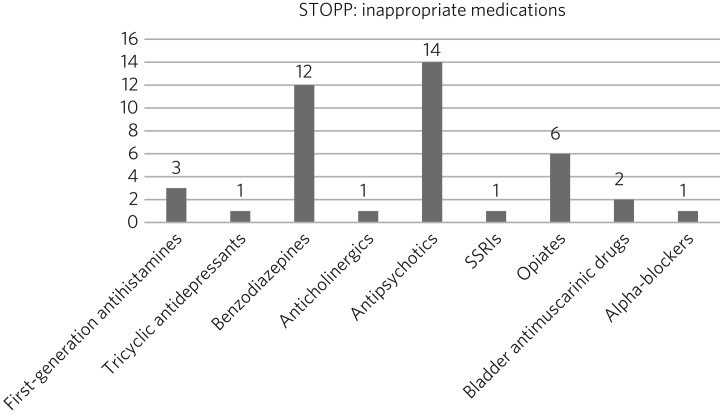
Inappropriate drugs prescribed according to STOPP.

**Fig. 2:**
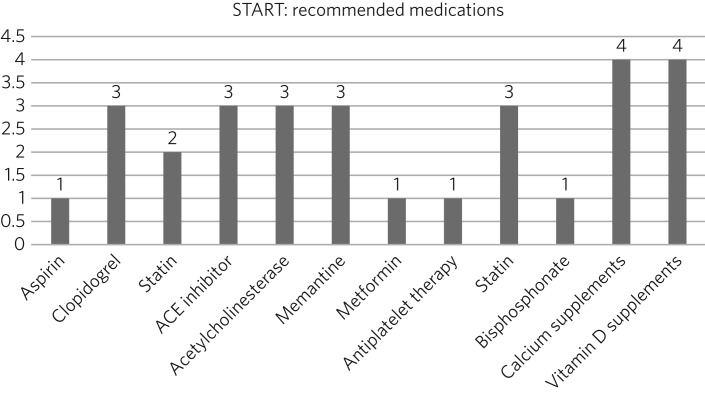
Drugs that should have been prescribed according to START.

Fig. 1 represents the STOPP part of the toolkit, while Fig. 2 represents the START component.

According to the STOPP toolkit, 164 drugs were prescribed; of those, 118 (72%) drugs were prescribed for longer than a year. Forty-one (25%) drugs were considered to have been inappropriately prescribed and could be stopped according to STOPP (Fig. 1). The four main types of inappropriateness were long-term use, lack of clear indication, symptoms no longer present, and lack of clear documentation. The most common drugs inappropriately prescribed were benzodiazepines, antipsychotics and opiates.

According to the START toolkit, 145 drugs were prescribed; of those, 129 (89%) drugs were prescribed for longer than a year. Twenty-six drugs should have been prescribed according to the toolkit but were not (Fig. 2).

## Discussion

When reviewing all geographical sectors, an improvement in the prescribing pattern was found between the audits. All teams were more conscious of inappropriate prescribing in older people. In some of the cases, we had little information on who prescribed the medications, why they were prescribed and when they were reviewed. Nevertheless, it is important to remember that the STOPP/START criteria were designed to highlight inappropriate prescriptions and prevent ADEs, especially those involving medications with a high risk of ADRs.

The audit showed an observed overall 24% reduction in polypharmacy and a 25% reduction in prescribing of medications with a high risk of ADRs. However, the difference between the audits was not statistically significant. The non-significance may be related to the reduction in number of patients from 58 to 47, or the reduction in drugs per patient may have been due to them having fewer illnesses.

According to the STOPP Toolkit, 164 drugs were prescribed in the 2016 audit. *This was a 25% reduction in prescribing since the first audit.* Seventy-five central nervous system (CNS) drugs were prescribed according to STOPP, a 40% reduction in prescribing since the first audit. According to the START toolkit, 145 drugs were prescribed at the time of the re-audit. *This was an 8% reduction in prescribing since the first audit.* There was no change in the total number of CNS drugs prescribed, according to the START toolkit. However, *there was less antidepressant prescribing across all sectors.* These results are also a proxy measure for improving care by optimising medicines in the elderly, i.e. reduced exposure to polypharmacy improves care and quality of life.

According to the toolkit, our CNS drugs will be mainly inappropriately prescribed because of long-term use (longer than a month). However, the nature of mental disorder and its associated behavioural and psychological symptoms will mean that it is appropriate for our patient groups to be on longer-term medication. The multiple comorbidities will also add to the complexity of those patients and their management.

Another important observation is that adequate documentation is very much needed to clarify the target symptoms and the rationale for prescribing. It is important for all the teams to review medications and their appropriateness as part of the weekly ward round and monthly clinical pharmacist input. It is important to continue to raise awareness of the STOPP/START tool and to encourage its use by doctors and pharmacists, in order to promote safe prescribing among older patients.

Prescribers should not feel overwhelmed when reviewing multiple medications prescribed for older people. The STOPP/START tool has been proven to be a useful framework.

The audit has provided the foundations of a good multidisciplinary relationship between medical, nursing and pharmacy staff, which has not only benefited the in-patients but also demonstrated how a multidisciplinary team can stop inappropriate prescribing in older patients. It is clear that we can improve the care and safety of such patients by optimising their medicines. This will also have a secondary economic impact by producing an annual cost saving. The additional cost benefits in preventing adverse effects and associated medical treatment should be included in any basic financial evaluation. These additional benefits are likely to be substantial in economic and human terms. The benefits of the medical and pharmaceutical perspectives working together as a team were both additive and synergistic.

The audit also highlighted the need to facilitate greater collaboration with a clinical pharmacist and the older person's physician/general practitioner to provide better care for older psychiatric patients. As the toolkit looked at the prescribing pattern, it does not include the patients’ views or their families’ opinion about drugs. However, shared decision-making should be always a priority for clinical teams. As the audit reflects a cross-section of old age psychiatry, we believe that the results are generalisable. It will be useful in the future for a well-designed research study to be conducted in old age psychiatry covering multiple areas or larger community and in-patient settings to test that assumption.

The small pocket-sized smartcards/leaflets showing the drugs commonly meeting the STOPP/START criteria proved useful on all wards. Introducing the toolkit at junior doctor induction has also been beneficial.

## Conclusion

Inappropriate prescribing occurs when the risks associated with prescribing a medication outweigh the potential benefits of the medication in a particular patient. PIP may also occur when a patient does not receive a medication indicated for the treatment or prevention of a disease or condition. The STOPP/START criteria have been used to review the medication profiles in various settings worldwide. STOPP/START criteria are validated, reliable systems-based criteria for PIP. The STOPP criteria significantly predict ADEs, and the application of the STOPP/START toolkit improves medication appropriateness and probably reduces/prevents adverse events. The STOPP/START criteria are *not* the complete answer to preventing medication errors, but they help to optimise pharmacotherapy at the point of initiation and at routine medication review. The use of the STOPP/START toolkit can also have a positive impact by reducing prescribing errors. Sustaining these changes will require continued efforts to maintain prescriber awareness of the STOPP/START toolkit.

## About the authors

**Dr Victor M. Aziz**, Consultant Psychiatrist, Cwm Taf UHB, Wales, UK; **Dr Natalie Hill**, ST6 in Old Age Psychiatry, Wales Deanery, UK; **Dr Sugandha Kumar**, ST6 in Old Age Psychiatry, Wales Deanery, UK.
